# Chemoradiation of locally advanced biliary cancer: A PRISMA‐compliant systematic review

**DOI:** 10.1002/cam4.70196

**Published:** 2024-12-10

**Authors:** Silvia Bisello, Claudio Malizia, Filippo Mammini, Erika Galietta, Federica Medici, Gian Carlo Mattiucci, Francesco Cellini, Andrea Palloni, Luca Tagliaferri, Gabriella Macchia, Francesco Deodato, Savino Cilla, Giovanni Brandi, Alessandra Arcelli, Alessio G. Morganti

**Affiliations:** ^1^ Radiation Oncology AOU delle Marche Ancona Italy; ^2^ Nuclear Medicine IRCCS Azienda Ospedaliero‐Universitaria di Bologna Bologna Italy; ^3^ Department of Medical and Surgical Sciences (DIMEC) Alma Mater Studiorum ‐ Bologna University Bologna Italy; ^4^ UOC Radioterapia Oncologica Mater Olbia Hospital Olbia Italy; ^5^ Dipartimento Universitario Diagnostica per Immagini, Radioterapia Oncologica ed Ematologia Università Cattolica del Sacro Cuore Rome Italy; ^6^ Dipartimento di Diagnostica per Immagini, Radioterapia Oncologica ed Ematologia Fondazione Policlinico Universitario “A. Gemelli” IRCCS Rome Italy; ^7^ Medical Oncology IRCCS Azienda Ospedaliero‐Universitaria di Bologna Bologna Italy; ^8^ Dipartimento di Diagnostica per Immagini, U.O.C. Radioterapia Oncologica, Radioterapia Oncologica Ed Ematologia Fondazione Policlinico Universitario Agostino Gemelli IRCCS Rome Italy; ^9^ Radiation Oncology Unit Gemelli Molise Hospital‐Università Cattolica del Sacro Cuore Campobasso Italy; ^10^ Medical Physics Unit Gemelli Molise Hospital Campobasso Italy; ^11^ Radiation Oncology IRCCS Azienda Ospedaliero‐Universitaria di Bologna Bologna Italy

**Keywords:** biliary tract cancers, brachytherapy, chemoradiation, literature review, systematic review

## Abstract

**Introduction:**

Biliary tract cancers (BTC) are rare and aggressive neoplasms. The current management of locally advanced or unresectable BTC is primarily based on chemotherapy (CHT) alone, linked to a median overall survival (OS) of approximately 12 months. However, international guidelines still consider concurrent chemoradiation (CRT) as an alternative treatment option. This study aims to review the current evidence on “modern” CRT for primary or recurrent unresectable BTC.

**Materials and Methods:**

A comprehensive search was conducted on PubMed, Scopus, and Cochrane Library to identify relevant papers. Prospective or retrospective trials reporting outcomes after concurrent CRT of unresectable non‐metastatic, primary, or recurrent BTC were included. Only English‐written papers published between January 2010 and June 2022 were considered.

**Results:**

Seventeen papers, comprising a total of 1961 patients, were included in the analysis. Among them, 11 papers focused solely on patients with primary unresectable BTC, while two papers included patients with isolated local recurrences and four papers encompassed both settings. In terms of tumor location, 12 papers included patients with intrahepatic, extrahepatic, and hilar BTC, as well as gallbladder cancer. The median CRT dose delivered was 50.4 Gy (range: 45.0–72.6 Gy) using conventional fractionation. Concurrent CHT primarily consisted of 5‐Fluorouracil or Gemcitabine. The pooled rates of 1‐year progression‐free survival (PFS) and OS were 40.9% and 56.2%, respectively. The median 1‐ and 2‐year OS rates were 63.1% and 29.4%, respectively. Grade ≥3 acute gastrointestinal toxicity ranged from 5.6% to 22.2% (median: 10.9%), while grade ≥3 hematological toxicity ranged from 1.6% to 50.0% (median: 21.7%).

**Conclusion:**

Concurrent CRT is a viable alternative to standard CHT in patients with locally advanced BTC, offering comparable OS and PFS rates, along with an acceptable toxicity profile. However, prospective trials are needed to validate and further explore these findings.

## Graphical abstract

This is a systematic review of the current evidence on “modern” chemoradiation for primary or recurrent unresectable biliary tract cancers. We have critically compared chemoradiation outcomes with those of other treatment options, seeking to determine whether specific chemoradiation modalities provide distinct advantages in terms of treatment efficacy and patient safety. This analysis is pivotal for informing future treatment guidelines and optimizing patient care in this challenging clinical area.

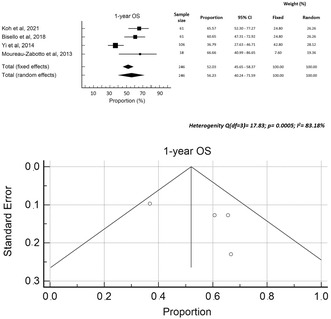

## 1 INTRODUCTION

Biliary tract cancers (BTC) represent a significant clinical challenge due to their rarity and aggressive nature, contributing to approximately 3% of all gastrointestinal cancers.^1^ These malignancies originate within the biliary tree, with classifications including intrahepatic cholangiocarcinoma (ICC), hilar cholangiocarcinoma (HCCA), extrahepatic cholangiocarcinoma (ECC), and gallbladder cancer (GBC). A key obstacle in the management of BTC is the frequent late‐stage diagnosis, which substantially limits treatment options and contributes to the dismally low 5‐year survival rates of 9%–16%.^2^ This underscores the urgent need for improved therapeutic strategies.

At present, the primary treatment for advanced BTC involves chemotherapy (CHT), primarily using a combination of gemcitabine and cisplatin.^3^, ^4^ This regimen is linked to a median survival period of approximately 12 months.^3^, ^4^ Additionally, there is growing interest in exploring systemic therapies targeting specific molecular pathways involved in BTC.^5^, ^6^ For cases that are unresectable or locally recurrent, international guidelines have proposed chemoradiation (CRT) as a viable alternative.^7^ In fact, the use of concurrent fluoropyrimidines‐ or gemcitabine‐based CRT has shown promising results in terms of both efficacy and tolerability.^8^, ^9^, ^10^, ^11^ However, there is a notable gap in the literature regarding optimal CRT target definition,^12^, ^13^ and comprehensive international guidelines for CRT in BTC are yet to be established. Furthermore, evidence on the use of CRT specifically for locally recurrent BTC remains limited and somewhat fragmented.^8^, ^14^, ^15^

One of the most critical voids in the current understanding is the lack of randomized trials that directly compare CHT and CRT in the context of locally advanced BTC. This leaves a significant question unanswered: does one treatment modality offer distinct advantages over the other? Moreover, the comparative efficacy and safety of CRT against other treatment modalities, such as best supportive care, stereotactic radiotherapy, and transarterial‐radioembolization, have not been sufficiently explored. Additionally, there is a scarcity of robust evidence guiding the optimal planning and delivery of CRT, including considerations for dose, fractionation, technique, and the integration of concurrent or adjuvant systemic therapies.

Given these gaps in knowledge, this study aims to conduct a comprehensive review of the existing literature on CRT in the context of primary or recurrent unresectable BTC. We will critically compare CRT outcomes with those of other treatment options, seeking to determine whether specific CRT modalities—such as dose and fractionation, radiotherapy techniques, drug combinations, radiotherapy boost, and target definition—provide distinct advantages in terms of treatment efficacy and patient safety. This analysis is pivotal for informing future treatment guidelines and optimizing patient care in this challenging clinical area.

## 2 MATERIALS AND METHODS

**FIGURE 1:**
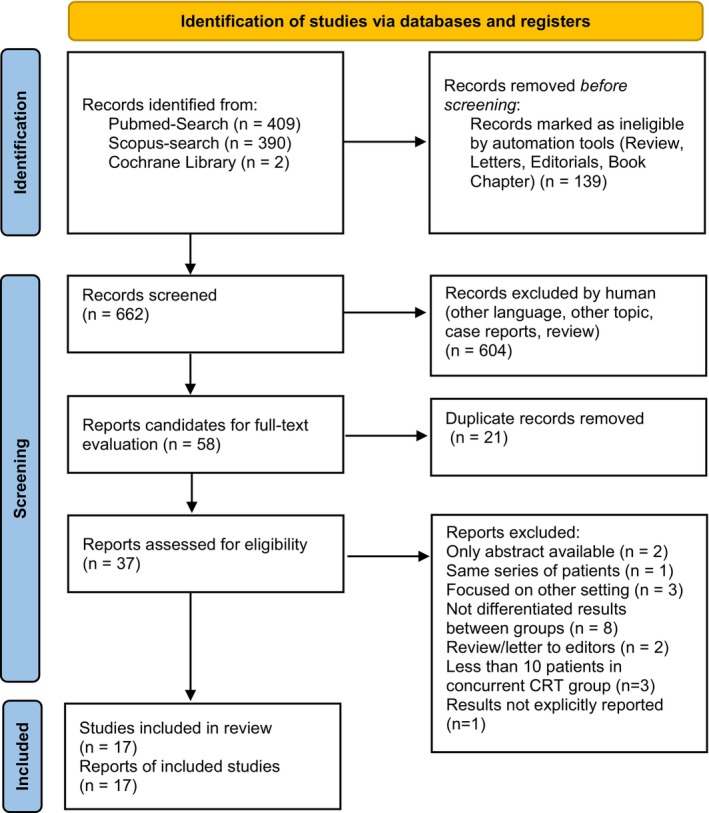
Flowchart of paper selection according to PRISMA 2020 diagram.

The protocol for this analysis was registered in the PROSPERO international prospective register of systematic reviews on July 17 2020.^16^ We followed the Preferred Reporting Items for Systematic Reviews and Meta‐Analysis (PRISMA) methodology.^17^ The flowchart of paper selection is shown in Figure 1.

### 2.1 Bibliographic Search

We conducted a literature search in PubMed, Scopus, and Cochrane Library. We included retrospective and prospective papers published from January 2010 to June 2022, reporting outcomes after concurrent CRT for primary or recurrent BTC. Only English‐written papers with a minimum of 10 patients treated with concurrent CRT were considered. The search used keywords such as “biliary tract neoplasms,” “biliary cancer,” “cholangiocarcinoma,” “radio‐CHT,” “chemo‐radiotherapy,” and “chemoradiation.” The complete search strings are shown in Supplementary Material A.

### 2.2 Inclusion Criteria

**FIGURE 2:**
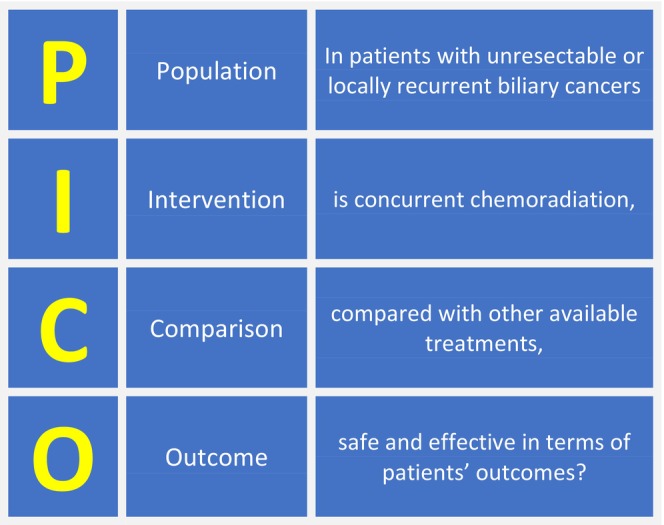
Research question framed in the PICO model.

Our research question was defined using the patient, intervention, comparison, outcome (PICO) model,^18^ as shown in Figure 2. The primary outcome was overall survival (OS), while secondary outcomes were progression‐free survival (PFS) and toxicity. Trials including metastatic patients or reporting on CRT in the adjuvant or neo‐adjuvant setting were excluded. Studies including patients with other abdominal cancers (hepatocellular carcinoma, ampullary or pancreatic adenocarcinoma) were excluded if the results were not differentiated based on the primary tumors. Systematic or narrative reviews, meta‐analyses, guidelines, book chapters, studies on animal models, preclinical studies, study protocols, and case reports were also excluded.

### 2.3 Study selection

Papers were independently screened by FMa and EG based on title and abstract. After removing duplicates, full‐text evaluation was independently performed by SB and FMe. Any disagreements were resolved by a third author (AGM). Papers excluded from full‐text evaluation with reasons for exclusion are listed in Supplementary material B.

### 2.4 Data analysis

Data on the included population (disease site and stage) and the delivered treatment (radiation dose and fractionation, any boost, concurrent CHT) were collected. Outcomes included median and/or 1‐ to 5‐year survival rates, median and/or 1‐ to 2‐year PFS rates, and acute and late toxicity rates. The outcome analysis, based on actuarial OS and PFS, was performed only for the CRT population. Values including other subgroups were listed as not reported (NR) or marked separately. A meta‐regression analysis was conducted between OS and radiation total dose and biologically effective dose (BED).

### 2.5 Quality assessment

We assessed the risk of bias using the ROBINS‐I tool (risk of bias in non‐randomized studies of intervention).^19^ Bias related to confounding factors, participant selection, intervention classification, deviations from intended intervention, missing data, outcome measurement, and selection of reported results were considered. Two authors (SB, FMa) independently ranked the included papers and resolved any disagreements through discussion. The results of this analysis were graphically reported using the robvis tool.^20^

## 3 RESULTS

### 3.1 Search results

A total of 17 papers were included in the analysis,^8^, ^10^, ^11^, ^14^, ^15^, ^21^, ^22^, ^23^, ^24^, ^25^, ^26^, ^27^, ^28^, ^29^, ^30^, ^31^, ^32^ comprising a total of 1961 patients. Among these studies, two were prospective trials,^21^, ^23^ while the rest were retrospective. The patients were treated between 1991 and 2018. Twelve studies focused on patients with primary unresectable non‐metastatic BTC,^10^, ^21^, ^22^, ^23^, ^25^, ^26^, ^27^, ^29^, ^30^, ^31^, ^32^ two studies included patients with isolated local recurrence,^15^, ^28^ and four studies considered both settings.^8^, ^11^, ^14^, ^24^ One study exclusively included patients with GBC,^30^ one study focused on ECC,^15^ one study analyzed only cases of HCCA,^22^ while two papers presented data on ICC.^31^, ^32^ The remaining papers included a mixed population of patients with various types of BTC. The stage of disease was reported in 15 studies,^8^, ^10^, ^11^, ^14^, ^15^, ^21^, ^22^, ^24^, ^25^, ^26^, ^27^, ^28^, ^29^, ^30^, ^32^ with a median of 69.6% of patients presenting with T3–4 tumor stage. The percentage of lymph node involvement was reported in 11 papers,^8^, ^10^, ^11^, ^14^, ^15^, ^22^, ^24^, ^26^, ^27^, ^28^, ^30^, ^32^ with a median of 46%. Table 1 provides further details on the characteristics of the patients. Five studies compared CRT with radiotherapy (RT),^11^, ^14^, ^15^, ^22^, ^28^ while four studies compared CRT with CHT.^23^, ^30^, ^31^, ^32^ One study compared definitive CRT to adjuvant and neoadjuvant CRT,^27^ one study compared CRT to best supportive care,^26^ and finally one study compared CRT to transarterial radioembolization or stereotactic RT.^29^ Among the publications reporting results on patients with locally advanced tumors, only one specified the version of the TNM classification used, which was the AJCC 6th edition.^26^ In the other publications, the stage classification was presented but the version of the TNM system used was not specified,^10^, ^21^, ^25^, ^29^, ^30^, ^31^, ^32^ while in other no data on the stage classification were provided.^22^, ^23^, ^27^

**TABLE 1:** Studies and patients characteristics.

Reference, year	Study design	N° patients included (total)	Treatment period	Diagnosis	Site (%)	T stage: 1–2 (%)	T stage: 3–4 (%)	cN1 (%)
Laughlin et al, 2022^27^	Retrosp.	29 (65)	1998–2019	UR	ECC: 90.0 HCCA: 10.0	64.0	36.0	38.0
Koh et al, 2021^28^	Retrosp.	61 (76)	2001–2015	LR	ECC: 57.0 HCCA: 43.0	58.0	42.0	29.0
Jethwa et al, 2020^10^	Retrosp.	48 (48)	1998–2018	UR	ECC: 85.0 GBC: 15.0	42.0	58.0	29.0
Hung et al, 2020^11^	Retrosp.	23 (30)	2015–2017	UR and LR	ICC: 60.0 ECC: 30.0 GBC: 10.0	26.7	70.0	56.7
Sebastian et al, 2019^29^	Retrosp.	54 (141)	2004–2014	UR	NR	51.9	48.1	NR
Bisello et al, 2018^8^	Retrosp.	61 (76)	1991–2017	UR and LR	ICC: 3.9 HCCA: 51.3 ECC: 32.9 GBC: 3.9 LR: 7.8	21.5	78.5	36.8
Verma et al, 2018^31^	Retrosp.	666 (2842)	2004–2013	UR	ICC: 100.0	NR	NR	NR
Verma et al, 2017^30^	Retrosp.	327 (1199)	2004–2013	UR	GBC: 100.0	7.0	59.0	30.0
Kim et al, 2017^15^	Retrosp.	18 (23)	2001–2013	LR	ECC: 100.0	30.4^a^	69.6^a^	47.8^a^
Jackson et al, 2016^32^	Retrosp.	374 (1636)	2001–2011	UR	ICC: 100.0	28.6^b^	71.3^b^	NR
Lee et al, 2016^21^	Prosp.	18 (18)	2007–2011	UR	ECC: 22.2 HCCA: 33.3 GBC: 44.5	22.2^b^	77.8^b^	NR
Chen et al, 2015^22^	Retrosp.	16 (34)	2001–2010	UR	HCCA: 100	31.0	69.0	50.0
Phelip et al, 2014^23^	Prosp. Phase II	18 (34)	2006–2010	UR	ICC: 56.0 ECC: 11.0 HCCA: 22.0 GBC: 11.0	NR	NR	NR
Moureau‐Zabotto et al, 2013^14^	Retrosp.	18 (30)	1995–2008	UR and LR	HCCA: 67.0 ECC: 33.3^b^	12.0^b^	75.0^b^	46.0^b^
Yoshioka et al, 2014^25^	Retrosp.	117 (498)	2000–2011	UR	ICCA: 14.0 ECC: 35.0 HCC: 44.0 GBC: 8.0^a^	53.0^a^	32.0^a^	NR
Yi et al, 2014^26^	Retrosp.	106 (176)	1995–2010	UR	ICC: 39.6 ECC: 29.2 GBC: 31.1	11.3	88.7	80.2
Habermehl et al, 2012^24^	Retrosp.	11 (25)	2003–2010	UR and LR	ECC: 36.4 HCCA: 63.6	0.0	100.0	81.8

Abbreviations: BTC, biliary tract cancer; ECC, extrahepatic cholangiocarcinoma; GBC, gallbladder cancer; HCCA, hilar cholangiocarcinoma (Klatskin tumor); ICC, intrahepatic cholangiocarcinoma; LR, local recurrence; NR, not reported; UR, unresectable.

^a^ Related to the whole population included in the analysis;

^b^ Stage reported according to American Joint Committee on Cancer (AJCC) staging system.

### 3.2 Treatment

The CRT targets were described in 10 papers.^8^, ^10^, ^11^, ^14^, ^15^, ^21^, ^22^, ^23^, ^25^, ^28^ Five studies^10^, ^11^, ^15^, ^22^, ^25^ defined the clinical target volume (CTV) as the sum of the gross tumor volume (GTV) and involved lymph nodes, while another five studies^8^, ^14^, ^21^, ^23^, ^28^ included the GTV and prophylactic nodal irradiation in the CTV. The planning target volume (PTV) was defined with an isometric expansion of the CTV by 10–20 mm in seven cases,^8^, ^14^, ^15^, ^22^, ^23^, ^25^, ^28^ and by 5 mm in two cases.^10^, ^21^ One study defined an internal target volume.^11^ Photon‐based RT was used in all studies except for one that used proton beams.^11^ The RT technique was reported in 11 papers,^8^, ^10^, ^14^, ^15^, ^22^, ^23^, ^24^, ^26^, ^27^, ^28^ with three‐dimensional conformal RT being used more frequently (10 studies),^8^, ^14^, ^15^, ^22^, ^23^, ^24^, ^26^, ^28^ while two studies used both three‐dimensional and intensity‐modulated RT techniques^10^, ^27^ and one study including also 2D technique (8). The median delivered dose was reported in 14 papers,^8^, ^10^, ^11^, ^14^, ^15^, ^21^, ^22^, ^23^, ^24^, ^25^, ^26^, ^27^, ^28^, ^29^ ranging from 45.0 to 72.6 Gy, with a median of 50.4 Gy. Conventional fractionation (1.8 or 2.0 Gy per fraction) was used in all studies^8^, ^10^, ^14^, ^15^, ^21^, ^22^, ^23^, ^24^, ^25^, ^26^, ^27^, ^28^, ^29^, ^33^ except for one series with patients receiving 72.6 Cobalt‐Gray Equivalent (CGE) in 3.3 CGE/fraction.^11^ A brachytherapy boost was delivered in five studies^8^, ^10^, ^24^, ^25^, ^27^ to a median of 17.0% of the study population, while two studies used an intraoperative RT boost to 2%–18% of the enrolled population.^24^, ^25^ The biologically effective dose (BED) ranged from 53.1 to 96.6 Gy (*α*/*β* = 10). Thirteen studies^8^, ^10^, ^11^, ^14^, ^15^, ^21^, ^22^, ^23^, ^24^, ^25^, ^26^, ^27^, ^28^ reported the concurrent CHT schedule, primarily based on 5‐fluorouracil or gemcitabine, with some studies also using capecitabine^8^, ^10^, ^15^, ^27^ or 5‐fluorouracil and leucovorin.^28^ Detailed treatment characteristics are provided in Table 2.

**TABLE 2:** Treatment characteristics.

Reference, year	CTV definition	PTV definition	RT‐technique	Median dose (range) Gy	Gy/Fraction	Boost %, Dose Gy	Median BED_α/ß10_ (range)	Comparisons	CHT schedule (%)
Laughlin et al, 2022^27^	NR	NR	3DRT IMRT	50.4 (7.2–62.4)	1.8–2.0	BRT 10.0% LDR 10.0(8.0–20.0)	59.5	nCRT vs. aCRT vs dCRT	5‐FU, CAPE
Koh et al, 2021^28^	GTV+PNI	CTV+10–20 mm	3DRT	54.0 (40.0–64.0)	1.8–3.0	No	64.8 (50.0–76.8)	CRT vs. RT	5‐FU, CAPE, leucovorin
Jethwa et al, 2020^10^	GTV+CIN+5–10 mm	CTV + 5–10 mm	3DRT IMRT	50.4 (45.0–50.4)	1.5–1.8	BRT 17.0% HDR 9.0 LDR 20.0–25.0	59.5	CRT±BRT boost^a^	5‐FU CAPE
Hung et al, 2020^11^	GTV+CIN+5 mm	ITV	PBT	72.6 (39.6–73.4) CGE	3.3	No	96.6 (52.7–109.6) CGE	CRT vs. RT	GEM 63.3 5‐FU 13.3^b^
Sebastian et al, 2019^29^	NR	NR	NR	50.4 (45.0–54.0)	1.8	No	59.5	SBRT vs. CRT vs. TARE	NR
Bisello et al, 2018^8^	GTV+PNI+10 mm	CTV+10 mm	2DRT 3DRT	50.0 (16.0–75.0)	1.8	BRT 51.3% 14.0 (14.0–50.0)	59.0	CRT±BRT boost	5‐FU 29.5 GEM 67.2 CAPE 3.3
Verma et al, 2018^31^	NR	NR	NR	NR	NR	No	NR	CRT vs. CHT	NR
Verma et al, 2017^30^	NR	NR	NR	NR	NR	No	NR	CRT vs. CHT	NR
Kim et al, 2017^15^	GTV+CNI+5–10 mm	CTV+10–20 mm	3DRT	54.0 (45.0–60.0)	1.8–2.0	No	63.7	CRT vs. RT	5‐FU 55.5, GEM 22.2, CAPE 16.6, Tegafur/uracil 5.5
Jackson et al, 2016^32^	NR	NR	NR	NR	NR	No	NR	CRT vs. CHT	NR
Lee et al, 2016^21^	GTV+PNI+bile duct	CTV+5 mm	3DRT	45.0	1.8	No	53.1	CRT+CHT	GEM + cisplatin 100.0
Chen et al, 2015^22^	GTV+CIN+5 mm	CTV+10 mm	3DRT	54.0 (40.0–66.6)^b^	1.8–2.0	Yes, not specified	63.7	CRT vs. RT	5‐FU 100.0
Phelip et al, 2014^23^	GTV+PNI	CTV+20 mm	3DRT	50.0	2.0	No	60.0	CRT vs. CHT	5‐FU+cisplatin
Moureau‐Zabotto et al, 2013^14^	GTV+PNI+15 mm	CTV+10 mm	3DRT	48.3 (30.0–78.0)	1.8–2.0	No	57.4	CRT vs. RT	5‐FU±cisplatin
Yoshioka et al, 2014^25^	GTV+/− PNI	CTV+10–20 mm	NR	50.0 (40.0–60.0)^b^	1.8–2.0	BRT 14.0%–18.0% IORT 2.0% 20.0–25.0^b^	59.0	RT±surgery±CHT	GEM 5‐FU
Yi et al, 2014^26^	NR	NR	3DRT	46.0 (36.0–52.0)	1.8–2.0	EBRT 100.0% 50.4 (45.0–60.0)	59.5	CRT vs. BSC	GEM 27.4 5‐FU 72.6
Habermehl et al, 2012^24^	NR	NR	3DRT	45.0 (39.0–50.4)^c^	1.8–2.0	BRT 18.2%‐24.0; IORT 18.2%‐24.0	53.1	Surgery+CRT vs RT or CRT±BRT/IORT boost	5FU 18.2 GEM 81.8

Abbreviations: 5‐FU 5‐fluorouracil, 3DRT 3D conformal radiotherapy; aCRT, adjuvant chemoradiation; BRT, brachytherapy; BSC, best supportive care; CAPE, capecitabine; CGE, cobalt Gray equivalent; CHT, chemotherapy; CHT‐RT, sequential chemo‐radiotherapy; CIN, clinically involved nodes; CRT, concurrent chemoradiation; CTV, clinical target volume; dCRT, definitive chemoradiation; GEM, gemcitabine; GTV, gross tumor volume; HDR, high dose rate brachytherapy; IMRT, intensity‐modulated radiotherapy; IORT, intra‐operative radiation therapy; ITV, internal target volume; LDR, low dose rate brachytherapy; nCRT, neoadjuvant chemoradiation; NR, not reported; PNI, prophylactic nodal irradiation; RT, radiotherapy; SBRT, stereotactic radiotherapy; TARE, trans‐arterial radioembolization.

^a^ CRT 94.0%, Prior CHT 4.0%, Adjuvant CHT 10.0%, BRT boost 17.0%;

^b^ Related to the whole population included in the analysis;

^c^ Two patients received 15 Gy with Intra‐operative radiotherapy, two patients received 24 Gy of brachytherapy boost.

### 3.3 Outcomes

**FIGURE 3:**
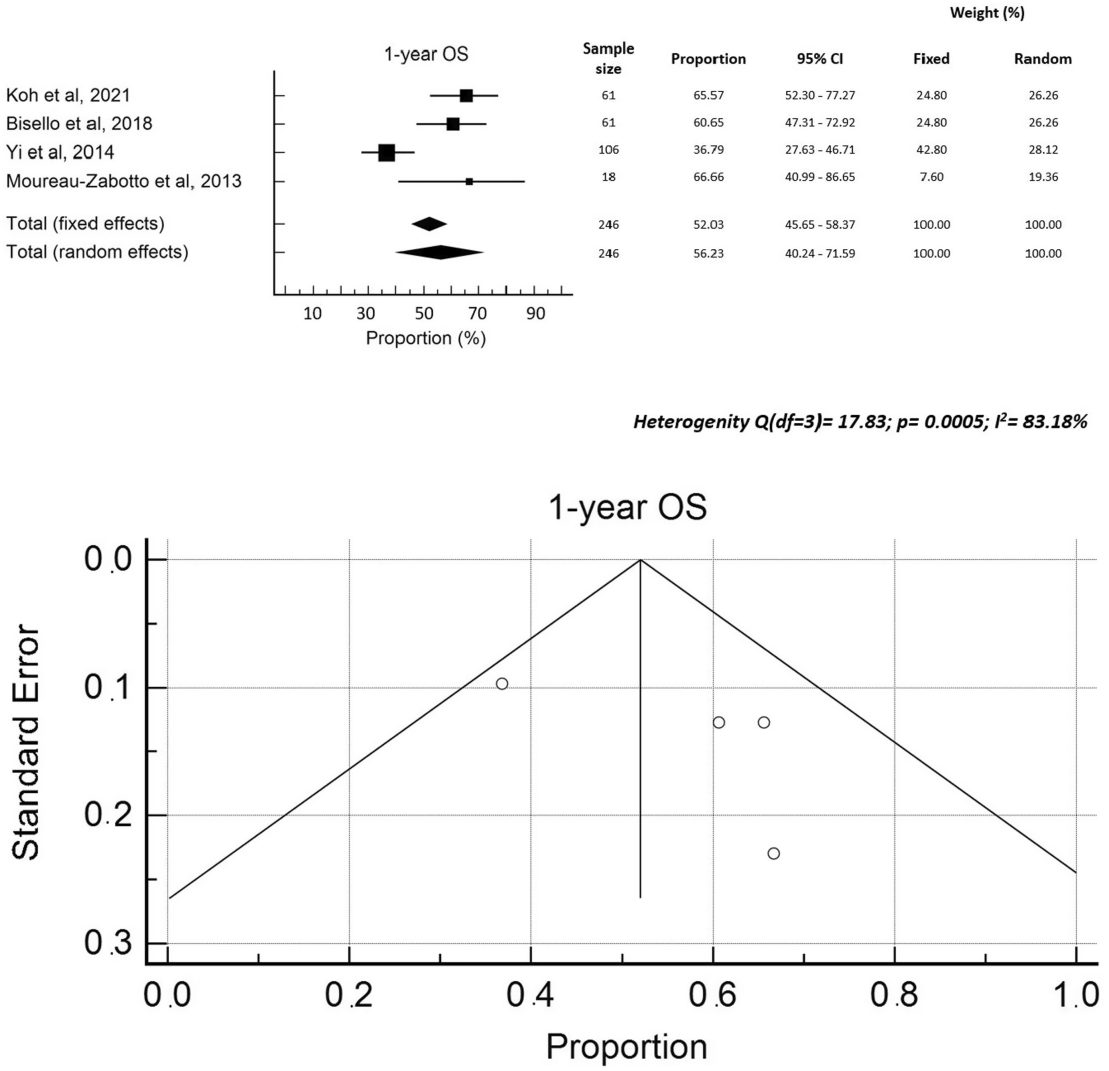
Forrest plot and funnel plot 1‐year OS.

**FIGURE 4:**
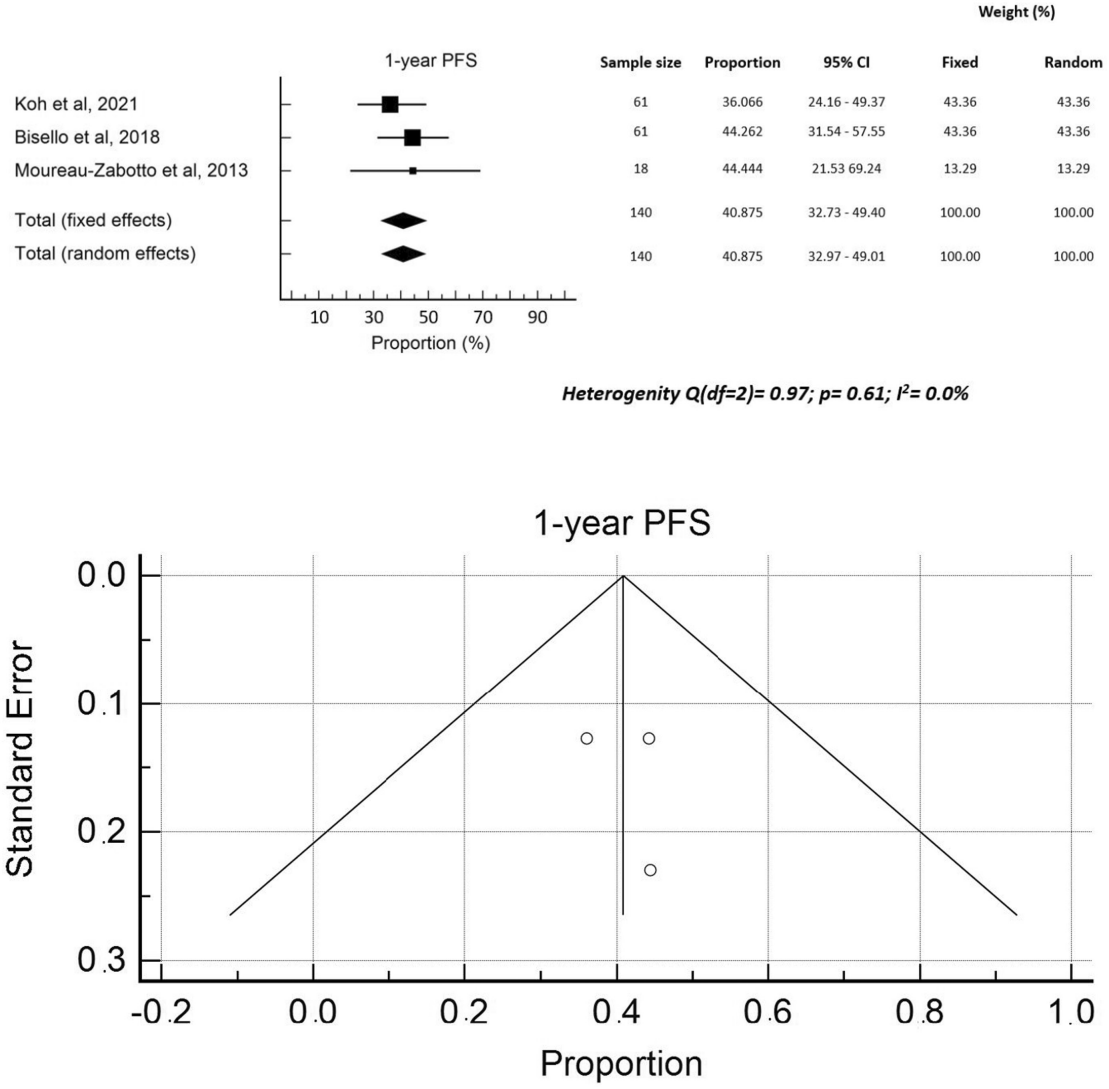
Forrest plot and funnel plot 1‐year PFS.

The median follow‐up was reported in 13 studies^10^, ^11^, ^14^, ^15^, ^22^, ^23^, ^24^, ^27^, ^28^, ^29^, ^30^, ^31^, ^32^ and ranged between 9.0 and 27.9 months (median 13.0 months). Median OS rates were reported in 13 studies^8^, ^10^, ^11^, ^21^, ^22^, ^23^, ^24^, ^25^, ^26^, ^29^, ^30^, ^31^, ^32^ and ranged between 9.6 and 20.0 months (median: 13.5 months). One‐year OS was reported in four^8^, ^14^, ^26^, ^28^ studies with rates ranging between 36.8% and 66.7% (median: 63.1%). Two‐year OS, reported in four studies,^8^, ^10^, ^15^, ^32^ ranged from 24.4% to 52.1% (median 29.4%). Two papers^10^, ^27^ reported 16.0%^27^ and 20.0%^10^ 3‐year OS rates, respectively, while three papers^10^, ^26^, ^27^ reported 5‐year OS rates, ranging from 0.0% to 7.9% (median: 1.9%). The median PFS was reported in six studies^10^, ^21^, ^22^, ^23^, ^24^, ^26^ with values ranging from 3.1 to 12.1 months (median: 8.2 months). Three papers^8^, ^14^, ^28^ reported one‐year PFS (median: 44.1%), while other three papers^8^, ^10^, ^15^ reported 2‐year PFS (median: 21.0%). A meta‐regression analysis was conducted on the impact of total dose and BED on OS, which showed no significant correlations, with a sample heterogeneity (*I* ^2^ test) of 55.8% and 54.0%, respectively. Finally, a Forrest plot and funnel plot of 1‐year OS ad 1‐year PFS were created ad are reported in Figure 3 and Figure 4, respectively. The heterogeneity test showed statistically significant values for OS but not for PFS. Moreover, an asymmetry is evident from the examination of the funnel plots, both for PFS and for OS, suggesting the possibility of publication bias. The pooled rates of 1‐year PFS and OS were 40.9% and 56.2%, respectively.

### 3.4 Response and treatment failures

Tumor response was assessed using the RECIST criteria^34^ in two studies.^21^, ^26^ A partial response was observed in 19.8%^26^ and 27.8%^21^ of patients, while stable disease was seen in 69.8%^26^ and 72.2%^21^ of patients, respectively. Two papers^11^, ^28^ reported data on local control (LC) with a 1‐year LC rate of 88.0%^11^ and freedom from local progression rate of 70.0%.^28^ Three other papers^10^, ^22^, ^27^ reported local failures as crude rates of 17.0% and 62.0%,^22^, ^27^ and a 2‐year rate of local progression of 27.0%.^10^ Distant metastases were reported as a crude value of 18.0%,^22^ and as a 2‐year rate of 33.0% in another study.^10^ Finally, distant progression was reported as a crude value of 38.0% in one study.^27^

### 3.5 Toxicity

Acute toxicity was scored according to the Common Terminology Criteria for Adverse Events (CTCAE), or the RTOG scale.^8^ Acute gastrointestinal toxicity (Grade ≥3) was registered in eight studies^8^, ^10^, ^11^, ^14^, ^21^, ^22^, ^23^, ^26^ and ranged from 5.6% to 13.2% (median 10.9%). Seven papers^8^, ^11^, ^21^, ^22^, ^23^, ^26^, ^28^ found Grade ≥3 acute hematologic toxicity, ranging from 1.6% to 50.0% (median 21.7%). Late toxicity was generally not reported. Treatment outcomes are summarized in Table 3.

**TABLE 3:** Outcomes.

Reference, year	Median FU, months (range)	Median OS, months, (range)	1‐y OS (%)	2‐y OS (%)	3‐ y OS (%)	5‐y OS (%)	Median PFS months, (range)	1‐y PFS (%)	2‐y PFS (%)	Tumor Response/Local Control (%)	Acute Toxicity G≥3, % (scale)	Findings
Laughlin et al, 2022^27^	9.0^a^	NR	NR	NR	16.0	0.0	NR	NR	NR	LP 17.0 DP 38.0	NRS (CTCAE 5.0)	Improved OS after nCRT + OLT compared to dCRT or aCRT
Koh et al, 2021^28^	13.0 (2.0–119.0)^a^	16.0 (13.0–19.0)^a^	66.0	NR	NR	NR	9.0 (7.0–11.0)^a^	37.0	NR	1‐y FFLP 70.0	HAE 1.6 (CTCAE 4.0)	Improved OS and PFS after CRT (compared to RT) or with BED >59.0 GY
Jethwa et al, 2020^10^	13.0 (6.0–29.0)	12.0 (2.3–73.2)	NR	33.0	20.0	7.0	9.0 (1.7–73.2)	NR	21.0	2‐ y LP 27.0 2‐y DM 33.0	GI 13.0 (CTCAE 4.0)	Improved OS and PFS with BED >59.5 Gy
Hung et al, 2020^11^	16.0 (3.0–36.0)	20.6	83.0^a^	32.0^a^	NR	NR	12.1	47.0^a^	NR	1‐y LC 88.0 1‐y DMF 68.0	GI 10.0 HAE 21.7 (CTCAE 4.0)	Improved OS, PFS, LC, and DMF after CRT compared to RT alone
Sebastian et al, 2019^29^	17.0^a^	14.0 (11.0–20.0)	NR	NR	NR	NR	NR	NR	NR	NR	NR	Improved OS after SBRT compared to CRT or TARE
Bisello et al, 2018^8^	NR	13.5	60.1	24.4	NR	NR	10.5	44.1	9.9	NR	GI 13.2 HAE 8.1 (RTOG)	Improved PFS after 2D‐CRT compared to 3D‐CRT
Verma et al, 2018^31^	10.0 (0–114.0)^a^	13.6 (12.3–15.7)	NR	NR	NR	NR	NR	NR	NR	NR	NR	Improved OS after CRT compared to CHT
Verma et al, 2017^30^	9.0 (0.0–123.0)^a^	12.9 (11.0–14.7)	NR	NR	NR	NR	NR	NR	NR	NR	NR	Improved OS after CRT compared to CHT
Kim et al, 2017^15^	14.2 (2.4–114.6)^a^	18.4 (4.4–114.6)^a^	62.6^a^	52.1	NR	NR	15.5 (1.6–114.6)^a^	56.3^a^	53.3	NR	(CTCAE 4.03) NR	Improved PFS after CRT compared to RT
Jackson et al, 2016^32^	11.3 (2.0–121.8)^a^	12.7	NR	25.8	NR	NR	NR	NR	NR	NR	NR	Improved OS after CRT compared to CHT
Lee et al, 2016^21^	NR	9.6 (5.4–30.4)	NR	NR	NR	NR	6.8 (4.5–19.8)	NR	NR	PR 27.8 SD 72.2	GI 5.6 HAE 50.0 (NCI CTC 4.0)	/
Chen et al, 2015^22^	9.4 (2.4–47.4)^a^	13.5 (9.4–17.7)	NR	NR	NR	NR	8.8 (5.2–10.7)	NR	NR	LP 62.0 DM 18.0^a^	GI 8.8 HAE 17.4^a^ (NCI CTC 3.0)	Improved OS and PFS with CRT compared to RT alone
Phelip et al, 2014^23^	27.9 (± 8.0)^a^	13.5 (7.8–22.6)	NR	NR	NR	NR	7.5 (2.8–12.5)	NR	NR	PD: 56.0	GI 11.8 HAE 23.0 (NCI‐CTC 2.0)	Similar PFS and OS after CRT or CHT
Moureau‐Zabotto et al, 2013^14^	12.0 (1.0–83.0)^a^	NRS	66.7 ± 11.1	NRS	NR	NR	NRS	44.4 ± 11.7	NRS	NRS	GI 22.0 Systemic 15.0 (NCI CTC 3.0)^b^	Similar OS after CRT compared to RT
Yoshioka et al, 2014^25^	NR	15.0 (12.0–17.0)	NR	NR	NR	NR	NR	NR	NR	NR	NR	Improved OS after surgery plus RT/CHT compared to CRT/CHT
Yi et al, 2014^26^	NR	10.5 (2.1–80.0)^c^	36.8	NR	NR	1.9	7.5 (5.7–9.2)^c^	NR	NR	PR 19.8 SD 69.8 PD 10.4	GI 9.4 HAE 21.7 (NCI CTC 3.0)	Improved OS after CRT compared to BSC
Habermehl et al, 2012^24^	13.0	13.6 (4.0–34.8)	NR	NR	NR	NR	3.1 (2.3–24.8)	NR	NR	NR	NRS	Improved OS after surgery plus CRT compared to CRT and RT
Median	13.0	13.5	63.1	29.4	18.0	1.9	8.2	44.1	21.0		GI 10.9 HAE 21.7	

BED biological equivalent dose; BSC best supportive care, CHT chemotherapy, CI confidence interval, CR complete response, CRT chemoradiation, DFS disease free survival, DM distant metastasis, DMF distant metastasis free; FFLP freedom from Local Progression; FU follow‐up, GI gastrointestinal, HAE hematological, HR hazard ratio, LP local Progression, NCI CTC National Cancer Institute–Common Toxicity Criteria, NR not reported, NRS not reported separately, OLT orthotopic liver transplant, OS overall survival, PD progressive disease, PFS progression‐free survival, PR partial response, RECIST Response Evaluation Criteria in Solid Tumors, RT radiotherapy, RTOG Radiation Therapy Oncology Group, SD stable disease, TARE trans‐arterial radioembolization.

^a^ Related to the whole population included in the analysis, therefore not included in the final calculation of the median;

^b^ Pain, fever, asthenia.

^c^ Median OS and PFS are express in weeks in the original paper (median OS 42.6 (8.3–320.1), median PFS 29.9 (22.9–36.8)).

### 3.6 Comparisons

Considering the series enrolling patients with a single tumor site, the median OS is 12.9, 13.5, 18.4, and 12.7–13.6 months in patients with ICC,^31^, ^32^ GBC,^30^ ECC,^15^ and HCC,^22^ respectively. In terms of tumor site, series enrolling only patients with local recurrence^15^, ^28^ showed higher median OS values (17.2 months; range 16.0–18.4 months) than those composed exclusively of patients with locally advanced disease (13.2 months; range: 9.6–15.0 months).^10^, ^21^, ^22^, ^23^, ^25^, ^26^, ^29^, ^30^, ^31^, ^32^ Four studies demonstrated a significant benefit in terms of PFS and OS with CRT compared to RT alone.^11^, ^15^, ^22^, ^28^ However, one retrospective study with a small sample size of only 18 patients treated with CRT found no differences.^14^ Three studies reported significantly improved OS with CRT compared to CHT alone,^30^, ^31^, ^32^ while a phase II trial with slow enrolment and only 18 patients treated with CRT showed no differences.^23^ Additionally, CRT was found to be superior to best supportive care,^26^ equivalent to transarterial radioembolization,^29^ and inferior to stereotactic RT^29^ in terms of OS. Notably, the superiority of stereotactic RT over CRT was observed in a series focusing only on ICC.^29^ Finally, two studies analyzing the impact of RT dose found that a BED >59 Gy^28^ or >59.5 Gy^10^ correlated with better outcomes in terms of PFS and OS.

### 3.7 Quality assessment

**FIGURE 5:**
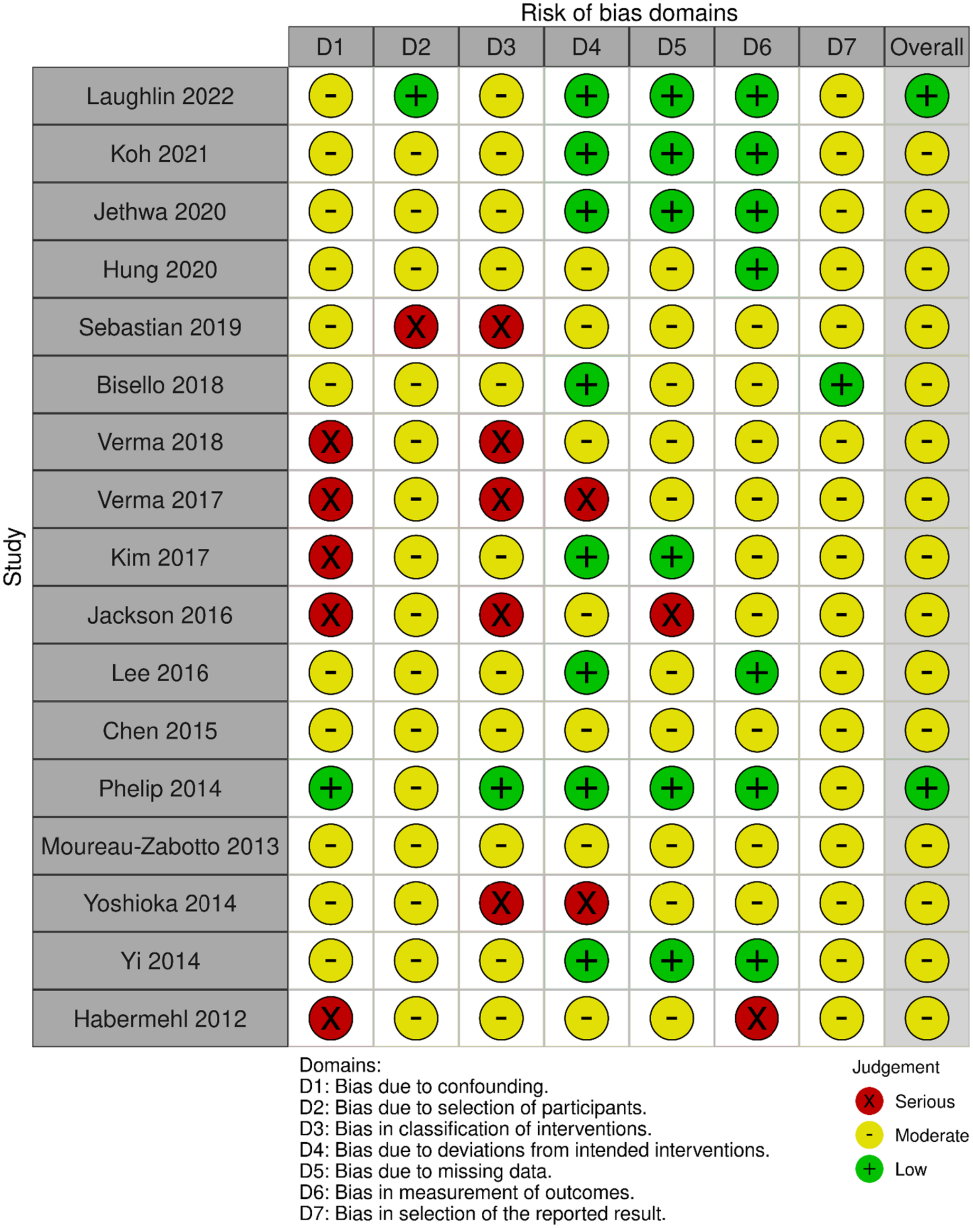
Risk of Bias in Non‐Randomized Studies—of Interventions (ROBINS‐I) traffic‐light plot.

**FIGURE 6:**
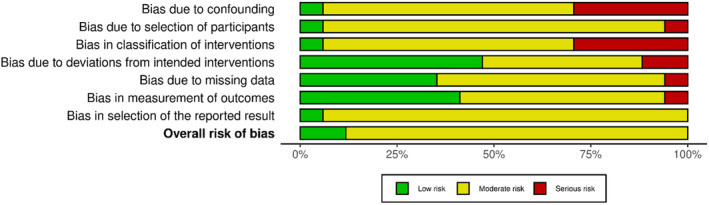
Risk of Bias in Non‐Randomized Studies—of Interventions (ROBINS‐I) summary plot.

Figures 5 and 6 display the traffic‐light plot and the summary plot based on the risk of bias in non‐randomized studies of intervention (ROBINS‐I) tool, respectively. The majority of the studies analyzed in this review had a moderate risk of bias, with only a few cases considered to have a serious risk. The domains that exhibited the highest risk of bias were “bias due to confounding” and “bias in classification of intervention.”

## 4 DISCUSSION

Our systematic literature review aimed to evaluate the differences between CRT and other treatments for locally advanced BTC. The key findings indicate that CRT offers promising results, with pooled rates of 1‐year PFS and OS being 40.9% and 56.2%, respectively. Notably, the incidence of grade ≥3 gastrointestinal toxicity was less than 15% across all studies, underscoring CRT viability as a treatment option for these tumors.^8^, ^10^, ^11^, ^14^, ^15^, ^21^, ^22^, ^23^, ^24^, ^25^, ^26^, ^27^, ^28^, ^29^, ^30^, ^31^

When comparing CRT with CHT, we observed different outcomes. One study reported similar results for both treatments, while three studies highlighted better OS following CRT.^23^, ^29^, ^30^, ^31^ Additionally, CRT seemed to offer superior PFS and OS compared to conventional fractionated RT in three studies.^11^, ^22^, ^28^ However, one study with a small sample size showed no significant differences.^14^ A separate study comparing CRT with stereotactic body radiation therapy (SBRT) reported better OS with SBRT, though it had certain limitations.^29^ This suggests that the choice between SBRT and CRT might depend on specific patient characteristics. For instance, ICC, located in the liver, might respond better to SBRT, whereas tumors near hollow organs or with regional lymph node metastases might benefit more from CRT.

Interestingly, the efficacy of CRT did not appear to be significantly influenced by the tumor site, as median survival was similar across ICC, GBC, and ECC.^20^, ^29^, ^31^, ^32^ Higher doses of CRT were associated with better outcomes in two studies,^10^, ^28^ yet our meta‐regression analysis did not show a significant effect of CRT dose on OS, likely due to limited variability in administered doses across the studies.

This study, however, has its limitations. Most included studies were retrospective, and there was an absence of randomized controlled trials, which limits the strength of our conclusions. The funnel plot analysis suggested a risk of publication bias, and the included studies were heterogeneous in terms of stage, tumor site, and treatment techniques. In particular, with respect to tumor site, it is notable that patients with ECC presented a median OS of 18.4 months,^15^ which appears superior to that of patients with ICC, GBC, and HCC (12.7–13.6 months^22^, ^30^, ^31^, ^32^). This variation underscores the importance of considering tumor site when evaluating outcomes and the potential benefits of treatment modalities. Furthermore, our analysis revealed heterogeneous survival outcomes between series that included only local recurrences^15^, ^28^ and those with only locally advanced tumors,^10^, ^21^, ^22^, ^23^, ^25^, ^26^, ^29^, ^30^, ^31^, ^32^ with a higher median survival observed in the former group (17.2 vs. 13.2 months). Interestingly, this heterogeneity was significant for OS but not for PFS, possibly due to the larger amount of data available for OS.

Furthermore, we must acknowledge that the previously reported comparisons between CRT and CHT are based on the evidence available during the period considered for analysis, when the standard CHT was represented by the combination of gemcitabine and cisplatin. However, two recent randomized trials have investigated the addition of immune checkpoint inhibitors (ICI) to standard CHT in advanced biliary cancer, demonstrating a modest but significant improvement in OS.^35^

The TOPAZ‐1 trial, with 685 patients, evaluated durvalumab (a PD‐L1 inhibitor) combined with gemcitabine and cisplatin. The results showed a significant improvement in OS with durvalumab (12.8 vs. 11.5 months; hazard ratio 0.80; *p* = 0.021), along with better PFS and objective response rate (ORR), with similar toxicity between groups.^36^

Similarly, the KEYNOTE‐966 trial studied pembrolizumab (a PD‐1 inhibitor) in 1069 newly diagnosed patients, also in combination with gemcitabine and cisplatin. Pembrolizumab significantly improved OS (12.7 vs. 10.9 months; hazard ratio 0.83; *p* = 0.0034) and PFS (6.5 vs. 5.6 months; *p* = 0.023) compared to placebo. Although the response rates were similar, the duration of response was longer with pembrolizumab. Survival benefits were consistent across all biliary cancer subtypes, and pembrolizumab did not significantly increase toxicity, maintaining health‐related quality of life.^37^

Unfortunately, it is challenging to compare the results of these studies with those in our review, as both studies enrolled both patients with locally advanced disease and metastatic patients. Furthermore, it should be noted that in both studies, patients with locally advanced disease were the minority (11.8%–13.9%) and that in one of the studies,^36^ no significant difference in terms of OS was recorded in the subgroup of non‐metastatic patients.

An important consideration in advanced BTC is that OS is often affected by complications like biliary obstructions and cholangitis, not just disease progression. This underscores the importance of considering variations in treatment approaches and the management of cancer‐related complications across different centers.

In conclusion, our analysis supports the potential role of CRT in inoperable BTC. However, there is a need for further research to identify patients who might benefit most from CRT, to confirm the impact of CRT dose on outcomes, and to determine the optimal treatment sequence. Considering the rarity of BTCs, conducting randomized studies in this field may be challenging. Alternative approaches like multi‐center data sharing and predictive modeling could be valuable in individualizing therapy based on patient characteristics.

## AUTHOR CONTRIBUTIONS

**Silvia Bisello:** Conceptualization (equal); data curation (equal); formal analysis (equal); investigation (equal); validation (equal); visualization (equal); writing – original draft (equal); writing – review and editing (equal). **Claudio Malizia:** Conceptualization (equal); formal analysis (equal); methodology (equal); validation (equal); visualization (equal); writing – original draft (equal); writing – review and editing (equal). **Filippo Mammini:** Conceptualization (equal); data curation (equal); validation (equal); visualization (equal); writing – original draft (equal); writing – review and editing (equal). **Erika Galietta:** Conceptualization (equal); data curation (equal); validation (equal); visualization (equal); writing – original draft (equal); writing – review and editing (equal). **Federica Medici:** Conceptualization (equal); validation (equal); visualization (equal); writing – original draft (equal); writing – review and editing (equal). **Gian Carlo Mattiucci:** Conceptualization (equal); validation (equal); visualization (equal); writing – original draft (equal); writing – review and editing (equal). **Francesco Cellini:** Conceptualization (equal); validation (equal); visualization (equal); writing – original draft (equal); writing – review and editing (equal). **Andrea Palloni:** Conceptualization (equal); validation (equal); visualization (equal); writing – original draft (equal); writing – review and editing (equal). **Luca Tagliaferri:** Conceptualization (equal); validation (equal); visualization (equal); writing – original draft (equal); writing – review and editing (equal). **Gabriella Macchia:** Conceptualization (equal); validation (equal); visualization (equal); writing – original draft (equal); writing – review and editing (equal). **Francesco Deodato:** Conceptualization (equal); validation (equal); visualization (equal); writing – original draft (equal); writing – review and editing (equal). **Savino Cilla:** Conceptualization (equal); formal analysis (equal); validation (equal); visualization (equal); writing – original draft (equal); writing – review and editing (equal). **Giovanni Brandi:** Conceptualization (equal); validation (equal); visualization (equal); writing – original draft (equal); writing – review and editing (equal). **Alessandra Arcelli:** Conceptualization (equal); validation (equal); visualization (equal); writing – original draft (equal); writing – review and editing (equal). **Alessio G. Morganti:** Conceptualization (equal); data curation (equal); formal analysis (equal); methodology (equal); validation (equal); visualization (equal); writing – original draft (equal); writing – review and editing (equal).

## FUNDING INFORMATION

This research received no external funding.

## CONFLICT OF INTEREST STATEMENT

Not declared.

## Supporting information


Data S1.

## Data Availability

All data supporting the reported results are included in this paper.
