# Supplementary material for: Chemoradiation of locally advanced biliary cancer: A PRISMA‐compliant systematic review
Source: Cancer Med. 2024 Dec 10;13(23):e70196. doi: 10.1002/cam4.70196 (PMC11632119; doi:10.1002/cam4.70196)
Supplement: Supplementary file 1 — Data S1. [file CAM4-13-e70196-s001.docx]

**SUPPLEMENTARY MATERIAL**

**Supplementary material A: keyword from database search**

Pubmed Search

"biliary tract neoplasms"[MeSH Terms] OR "biliary tract neoplasms" OR "biliary cancer"[All Fields] OR "cholangiocarcinoma"[All Fields]) AND ("chemoradiotherapy"[MeSH Terms] OR "chemoradiation"[All Fields] OR "chemoradiotherapy"[All Fields])

Scopus Search

( TITLE-ABS-KEY ( "biliary tract neoplasms" OR "biliary tract neoplasms" OR "biliary cancer" OR "cholangiocarcinoma" ) AND TITLE-ABS-KEY ( "chemoradiotherapy" OR "chemoradiation" ) )

Cochrane Library Search

( TITLE-ABS-KEY ( "biliary cancer") AND TITLE-ABS-KEY ( "chemoradiation" ) )

**Supplementary material B: papers excluded from full-text evaluation and reasons**

1. H.N. Yankey, K. Ruth, J.E. Meyer. Survival and Toxicity in Patients With Unresectable/Inoperable Biliary Cancers Treated with SBRT vs. Conventionally Fractionated Chemoradiation. International Journal of Radiation Oncology Biology Physics, 2021. 10.1016/j.ijrobp.2021.07.456. (abstract)
2. J. Yue, R. Feng. Efficacy and Safety of PD-1 Inhibitor SHR-1210 With Chemoradiotherapy in Locally Advanced Cholangiocarcinoma. International Journal of Radiation Oncology Biology Physics, 2021. 10.1016/j.ijrobp.2021.07.458. (abstract)
3. M. Oertel, F. Gattermann, H. Schmidt, HT Eich. Examining the Use of Radiation Therapy for Cholangiocarcinoma: Benefits through Modern Techniques. Oncology Research and Treatment, 2021: 44(7-8):408-413. 10.1159/000517533 (Less than 10 patients treated with concurrent CRT)
4. K. Morino, S. Seo, T. Yoh, K. Fukumitsu, T. Ishii, K. Taura, S. Morita, T. Kaido, S. Uemoto. Proposed Definition for Oligometastatic Recurrence in Biliary Tract Cancer Based on Results of Locoregional Treatment: A Propensity-Score-Stratified Analysis. Annals of Surgical Oncology, 2020: 27(6):1908-1917. 10.1245/s10434-020-08207-0. (Results non differentiated between groups)
5. A. Torgeson , S. Lloyd, D. Boothe, G. Cannon, I. Garrido-Laguna, J. Whisenant, M. Lewis, R. Kim, C. Scaife, R. Tao. Chemoradiation Therapy for Unresected Extrahepatic Cholangiocarcinoma: A Propensity Score-Matched Analysis. Annals of Surgical Oncology, 2017: 24(13):4001-4008. 10.1245/s10434-017-6131-9. (Results non differentiated between groups; included ampullary cancers).
6. J. Il Yu, H. Chul Park, D. Hoon Lim, J. Oh Park, Y. Suk Park, S. Tae Kim, S. Ho Choi, D Wook Choi, I. Woong Han, J. Seok Heo. Clinical outcomes of salvage chemoradiotherapy for locally recurrent biliary tract cancer. Tumori, 2017: 103(4):345-352. 10.5301/tj.5000666. (Ampullary cancers included and results not differentiated between groups)
7. EL. Pollom, M. Alagappan, LS Park, AS Whittemore, AC Koong, DT Chang. Does radiotherapy still have a role in unresected biliary tract cancer? Cancer Medicine, 2017: 6(1):129-141. 10.1002/cam4.975. (Included metastatic patients and results not divided between groups)
8. R. Autorino, GC. Mattiucci, F. Ardito, M. Balducci, F. Deodato, G. Macchia, G. Mantini, V. Perri, A. Tringali, MA. Gambacorta, L. Tagliaferri, F. Giuliante, AG. Morganti, V. Valentini. Radiochemotherapy with Gemcitabine in Unresectable Extrahepatic Cholangiocarcinoma: Long-term Results of a Phase II Study. Anticancer research, 2016: 36(2):737-40. (data reported also in a subsequent paper)
9. HM. Kim, KJ. Lee, J. Cha, MJ Chung, S. Bang, J. Seong, SY. Song, SW. Park. A pilot study of S-1-based concurrent chemoradiotherapy in patients with biliary tract cancer. Cancer Chemotherapy and Pharmacology, 2014: 74(4):861-5. 10.1007/s00280-014-2565-y. (Included patients treated with radical surgery and results not divided between groups)
10. C. Makita, T. Nakamura, A. Takada, K. Takayama, M. Suzuki, Y. Ishikawa, Y. Azami, T. Kato, I. Tsukiyama, Y. Kikuchi, M. Hareyama, M. Murakami, N. Fuwa, M. Hata, T. Inoue. Clinical outcomes and toxicity of proton beam therapy for advanced cholangiocarcinoma. Radiation Oncology, 2014: 14;9:26. 10.1186/1748-717X-9-26. (Less than 10 patients underwent concurrent chemoradiation)
11. YI. Kim, JW. Park, BH. Kim, SM. Woo, TH. Kim, YH. Koh, WJ. Lee, CM. Kim. Outcomes of concurrent chemoradiotherapy versus chemotherapy alone for advanced-stage unresectable intrahepatic cholangiocarcinoma. Radiation Oncology, 2013: 21;8:292. 10.1186/1748-717X-8-292. (metastatic patients included and results not differentiated)
12. K. Chaiyasit, V. Wiwanitkit. Unsectable cholangiocarcinoma: Additional therapy to chemoradiotherapy. Indian Journal of Medical and Paediatric Oncology, 2013: 34(1):49. 10.4103/0971-5851.113435. (Letter to editor)
13. E. Leong, WW. Chen, E. Ng, G. Van Hazel, A. Mitchell, N. Spry. Outcomes from combined chemoradiotherapy in unresectable and locally advanced resected cholangiocarcinoma. Journal of Gastrointestinal Cancer, 2012: 43(1):50-5. 10.1007/s12029-010-9213-5. (Only adjuvant setting considered)
14. AP. Ghafoori, JW. Nelson, CG. Willett, J. Chino, DS. Tyler, HI. Hurwitz, HE. Uronis, MA. Morse, RW. Clough, BG. Czito. Radiotherapy in the treatment of patients with unresectable extrahepatic cholangiocarcinoma. Int J Radiat Oncol Biol Phys 2011 Nov 1;81(3):654-9. (data on chemoradiation not reported separately)
15. V. Verma, CH. Crane. Contemporary perspectives on the use of radiation therapy for locally advanced gallbladder cancer. Chinese Clinical Oncology, 2019: 8(4):41. 10.21037/cco.2019.08.12. (Review)
16. T. Sumiyoshi, Y. Shima, T. Okabayashi, Y. Negoro, Y. Shimada, J. Iwata, M. Matsumoto, Y. Hata, Y. Noda, K. Sui, T. Sueda. Chemoradiotherapy for Initially Unresectable Locally Advanced Cholangiocarcinoma. World J Surg. 2018 Sep;42(9):2910-2918 (Only neoadjuvant setting considered)
17. BH. Kim, K. Kim, EK. Chie, J. Kwon, JY. Jang, SW. Kim, DY. Oh, YJ. Bang. Risk stratification and prognostic nomogram for post-recurrence overall survival in patients with recurrent extrahepatic cholangiocarcinoma. HPB (Oxford). 2017 May;19(5):421-428 (Results not differentiated between groups)
18. R. Dhanasekaran, AW. Hemming, I. Zendejas, T. George, DR. Nelson, C. Soldevila-Pico, RJ. Firpi, G. Morelli, V. Clark, R. Cabrera. Treatment outcomes and prognostic factors of intrahepatic cholangiocarcinoma. Oncology Reports, 2013: 29(4):1259-67. 10.3892/or.2013.2290. (Only adjuvant and palliative setting considered)
19. S. Sinha, R. Engineer, V. Ostwal, A. Ramaswamy, S. Chopra, N. Shetty. Radiotherapy for locally advanced unresectable gallbladder cancer - A way forward: Comparative study of chemotherapy versus chemoradiotherapy. J Cancer Res Ther. Jan-Mar 2022;18(1):147-151. (Less than 10 patients treated with CRT)
20. Chang WW, Hsiao PK, Qin L, Chang CL, Chow JM, Wu SY. Treatment outcomes for unresectable intrahepatic cholangiocarcinoma: Nationwide, population-based, cohort study based on propensity score matching with the Mahalanobis metric. Radiother Oncol 2018. (Resultes not explicitly reported, but only in terms of HR).

**Supplementary material 3**: PRISMA checklist

| **Section/topic** | **#** | **Checklist item** | **Reported on page #** |
| --- | --- | --- | --- |
| **TITLE** | | |  |
| Title | 1 | Identify the report as a systematic review, meta-analysis, or both. | 1 |
| **ABSTRACT** | | |  |
| Structured summary | 2 | Provide a structured summary including, as applicable: background; objectives; data sources; study eligibility criteria, participants, and interventions; study appraisal and synthesis methods; results; limitations; conclusions and implications of key findings; systematic review registration number. | 2 |
| **INTRODUCTION** | | |  |
| Rationale | 3 | Describe the rationale for the review in the context of what is already known. | 3 |
| Objectives | 4 | Provide an explicit statement of questions being addressed with reference to participants, interventions, comparisons, outcomes, and study design (PICOS). | Fig. 2 |
| **METHODS** | | |  |
| Protocol and registration | 5 | Indicate if a review protocol exists, if and where it can be accessed (e.g., Web address), and, if available, provide registration information including registration number. | 3 |
| Eligibility criteria | 6 | Specify study characteristics (e.g., PICOS, length of follow-up) and report characteristics (e.g., years considered, language, publication status) used as criteria for eligibility, giving rationale. | 3 |
| Information sources | 7 | Describe all information sources (e.g., databases with dates of coverage, contact with study authors to identify additional studies) in the search and date last searched. | 3 |
| Search | 8 | Present full electronic search strategy for at least one database, including any limits used, such that it could be repeated. | Suppl. Mat. A |
| Study selection | 9 | State the process for selecting studies (i.e., screening, eligibility, included in systematic review, and, if applicable, included in the meta-analysis). | 4 |
| Data collection process | 10 | Describe method of data extraction from reports (e.g., piloted forms, independently, in duplicate) and any processes for obtaining and confirming data from investigators. | 4 |
| Data items | 11 | List and define all variables for which data were sought (e.g., PICOS, funding sources) and any assumptions and simplifications made. | 4 |
| Risk of bias in individual studies | 12 | Describe methods used for assessing risk of bias of individual studies (including specification of whether this was done at the study or outcome level), and how this information is to be used in any data synthesis. | 4 |
| Summary measures | 13 | State the principal summary measures (e.g., risk ratio, difference in means). | 4 |
| Synthesis of results | 14 | Describe the methods of handling data and combining results of studies, if done, including measures of consistency (e.g., I^2^) for each meta-analysis. | 4 |

| **Section/topic** | **#** | **Checklist item** | **Reported on page #** |
| --- | --- | --- | --- |
| Risk of bias across studies | 15 | Specify any assessment of risk of bias that may affect the cumulative evidence (e.g., publication bias, selective reporting within studies). | 4 |
| Additional analyses | 16 | Describe methods of additional analyses (e.g., sensitivity or subgroup analyses, meta-regression), if done, indicating which were pre-specified. | 4 |
| **RESULTS** | | |  |
| Study selection | 17 | Give numbers of studies screened, assessed for eligibility, and included in the review, with reasons for exclusions at each stage, ideally with a flow diagram. | Fig. 1, Suppl. Mat. B |
| Study characteristics | 18 | For each study, present characteristics for which data were extracted (e.g., study size, PICOS, follow-up period) and provide the citations. | Tables 1,2,3 |
| Risk of bias within studies | 19 | Present data on risk of bias of each study and, if available, any outcome level assessment (see item 12). | Figures 5,6 |
| Results of individual studies | 20 | For all outcomes considered (benefits or harms), present, for each study: (a) simple summary data for each intervention group (b) effect estimates and confidence intervals, ideally with a forest plot. | Figures 3,4 |
| Synthesis of results | 21 | Present results of each meta-analysis done, including confidence intervals and measures of consistency. | Figures 3,4 |
| Risk of bias across studies | 22 | Present results of any assessment of risk of bias across studies (see Item 15). | Figures 5,6 |
| Additional analysis | 23 | Give results of additional analyses, if done (e.g., sensitivity or subgroup analyses, meta-regression [see Item 16]). | 5 |
| **DISCUSSION** | | |  |
| Summary of evidence | 24 | Summarize the main findings including the strength of evidence for each main outcome; consider their relevance to key groups (e.g., healthcare providers, users, and policy makers). | 6 |
| Limitations | 25 | Discuss limitations at study and outcome level (e.g., risk of bias), and at review-level (e.g., incomplete retrieval of identified research, reporting bias). | 6 |
| Conclusions | 26 | Provide a general interpretation of the results in the context of other evidence, and implications for future research. | 6 |
| **FUNDING** | | |  |
| Funding | 27 | Describe sources of funding for the systematic review and other support (e.g., supply of data); role of funders for the systematic review. | 1 |

N/A: not applicable
